# Supplementary material for: A crucial role for dynamic expression of components encoding the negative arm of the circadian clock
Source: Nat Commun. 2023 Jun 8;14:3371. doi: 10.1038/s41467-023-38817-7 (PMC10250352; doi:10.1038/s41467-023-38817-7)
Supplement: Supplementary file 1 — Supplementary Information [file 41467_2023_38817_MOESM1_ESM.pdf]

Supplementary Figure 1

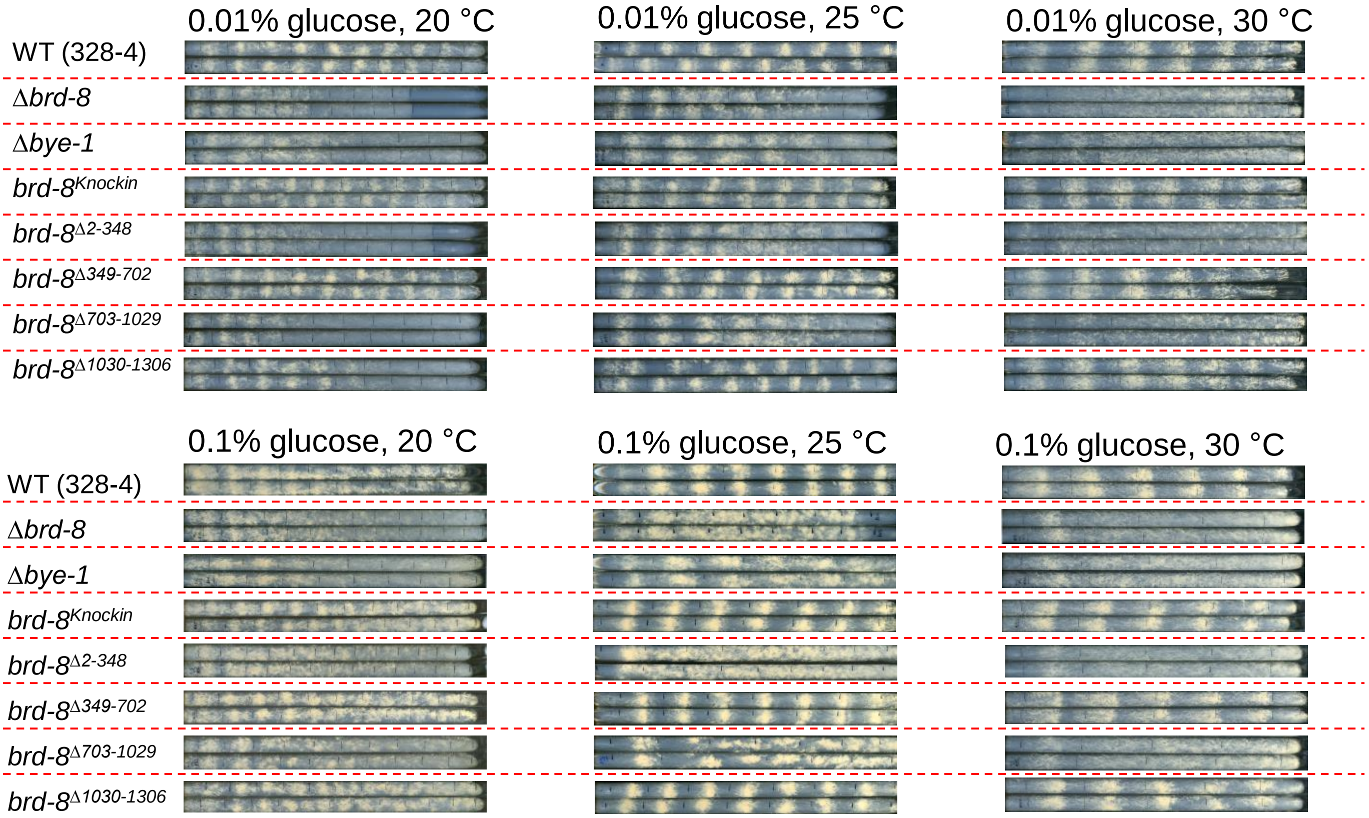

Supplementary Figure 1 Race tube analyses of Δ*brd-8*, Δ*bye-1*, and *brd-8* mutants deleting indicated regions. The deletion or mutant strains were backcrossed to *ras-1*^{bd} to facilitate visualization of circadian outputs. Race tube medium contains 0.1% or 0.01% glucose and cultures were incubated at 20, 25, or 30 °C as indicated. See Methods for details.

Supplementary Figure 2

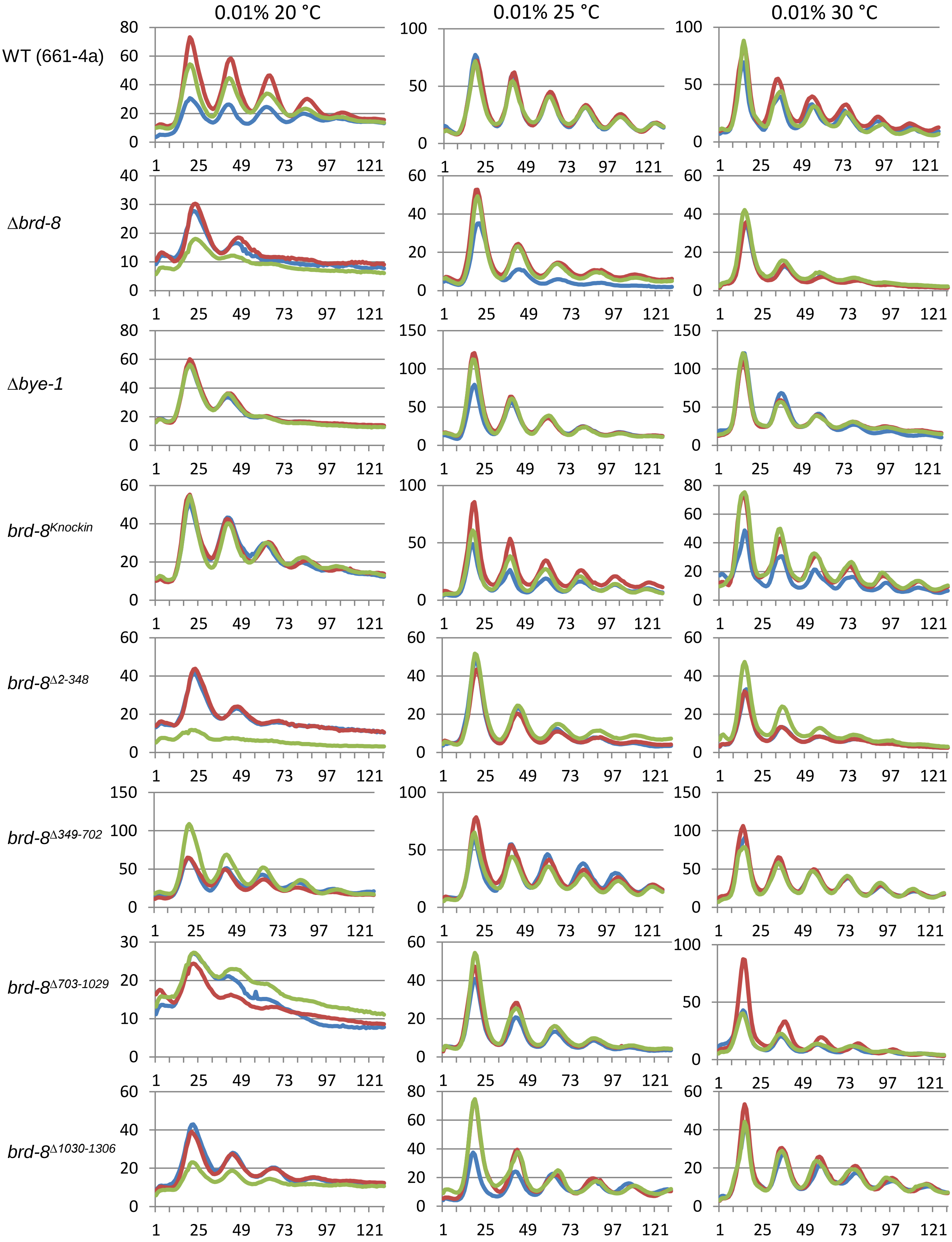

Supplementary Figure 2 continued

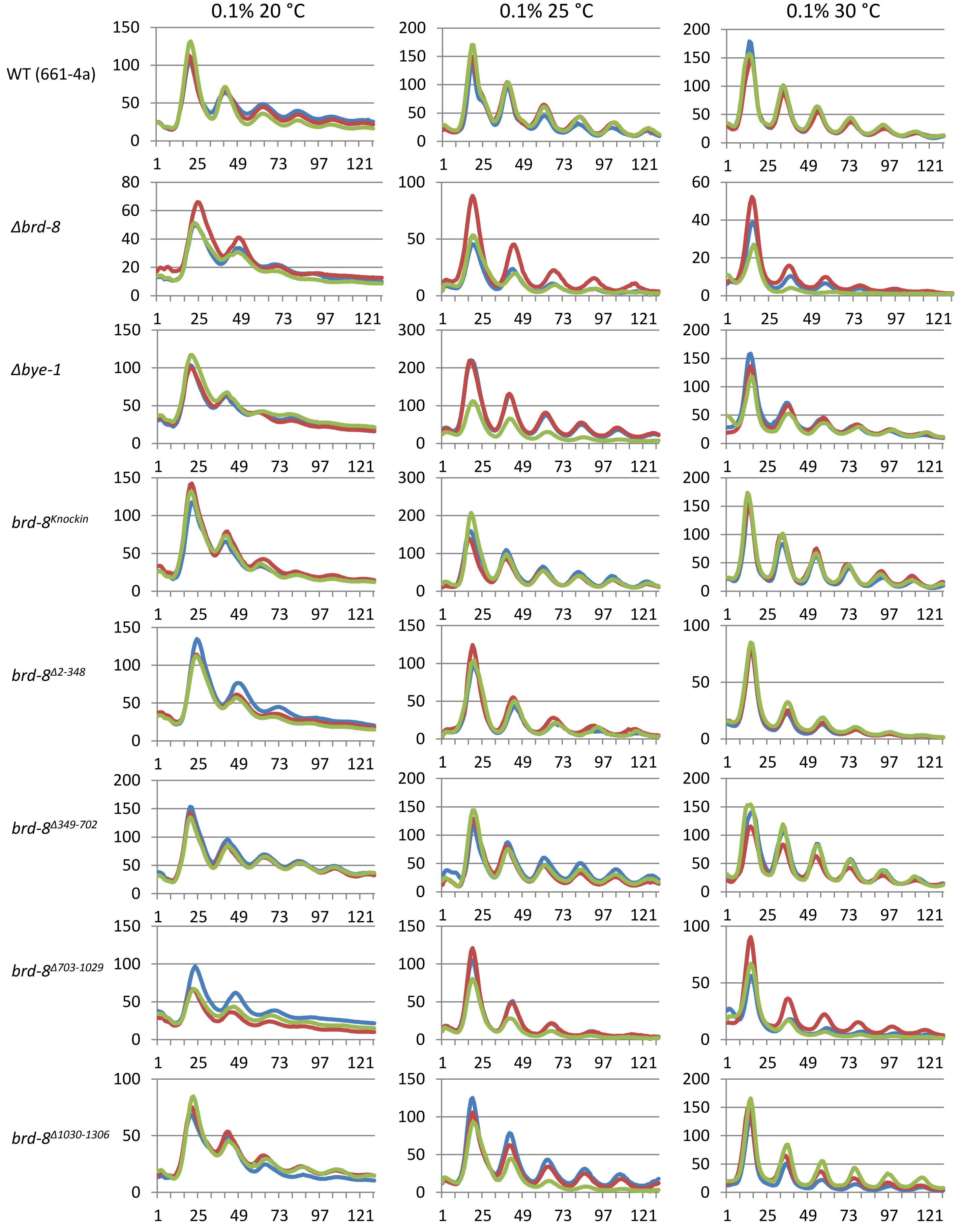

Supplementary Figure 2 continued

Supplementary Figure 2 Luciferase analyses of Δ*brd-8*, Δ*bye-1*, and *brd-8* mutants. Strains were cultured in 96 well plates bearing race tube medium with 0.1% or 0.01% glucose and incubated at 20, 25, or 30 °C as indicated. Bioluminescent signals were recorded by a CCD-camera every hour, the data were obtained using ImageJ with a custom macro, and circadian period lengths were manually determined. Raw data from three replicates were plotted with the X-axis (time (hrs)) and Y-axis (arbitrary units) respectively.

Supplementary Figure 3

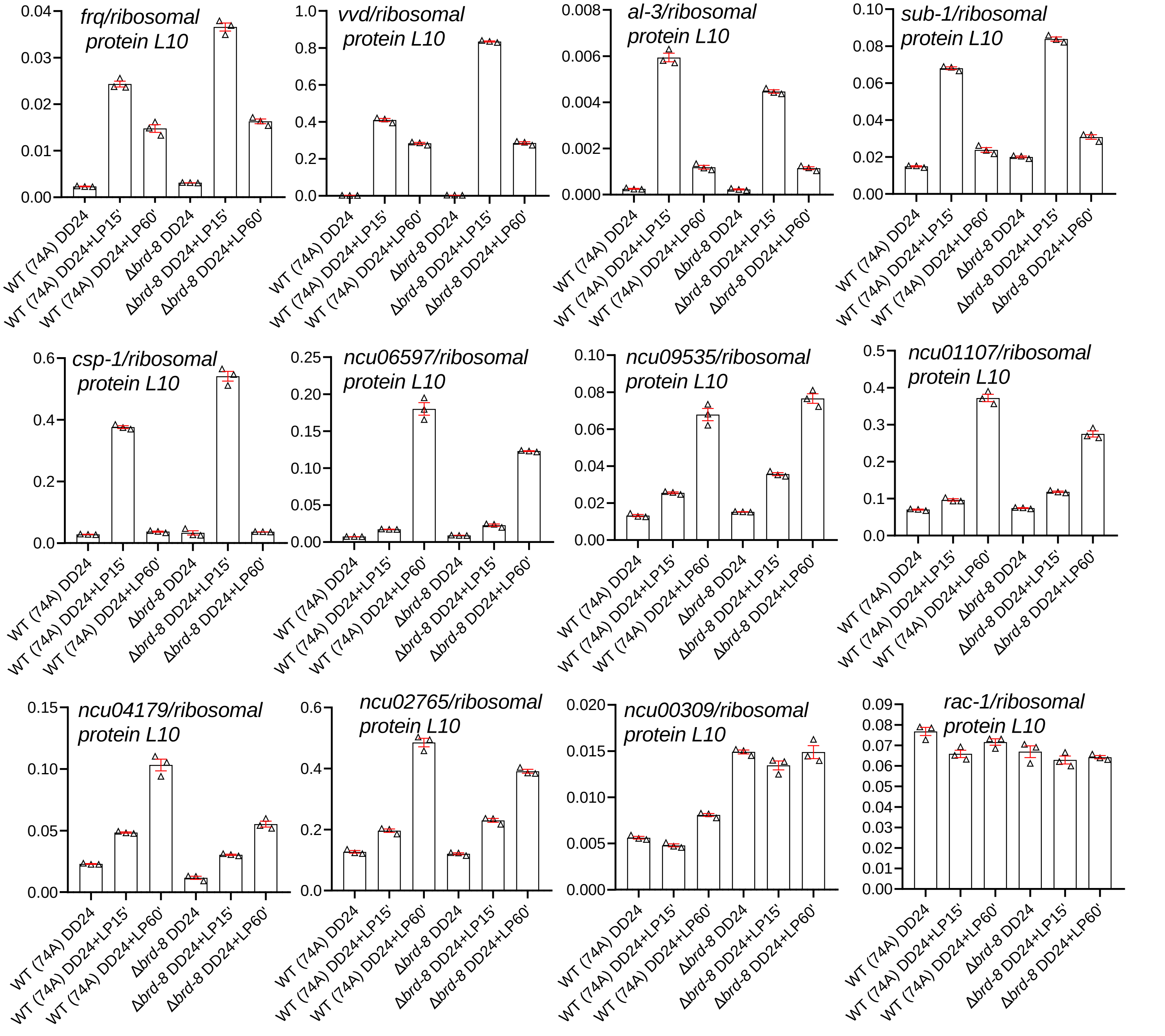

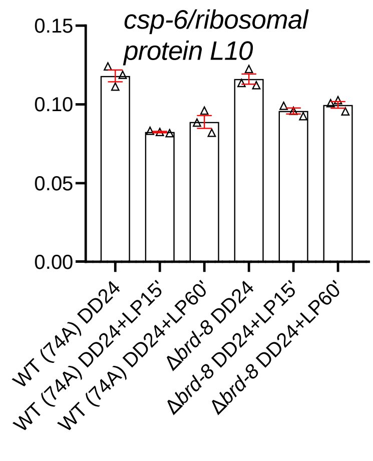

Supplementary Figure 3 Expression of light responsive genes in WT and Δ*brd-8*. mRNA levels of indicated genes in WT and Δ*brd-8* were assayed by RT-qPCR with samples grown under dark for 24 hrs or treated with a 15- or 60-min light exposure after 24 hrs in the dark as indicated. Expression of these genes was normalized to that of ribosomal protein L10 (*ncu08964*). For RT-qPCR assays, three technical replicates were performed, and the bars represent average values from qPCR reactions for indicated genes normalized to those of *L10* (*ncu08964*), with error bars representing SEMs (n= 3). Source data were stored in the Source Data file.

Supplementary Figure 4

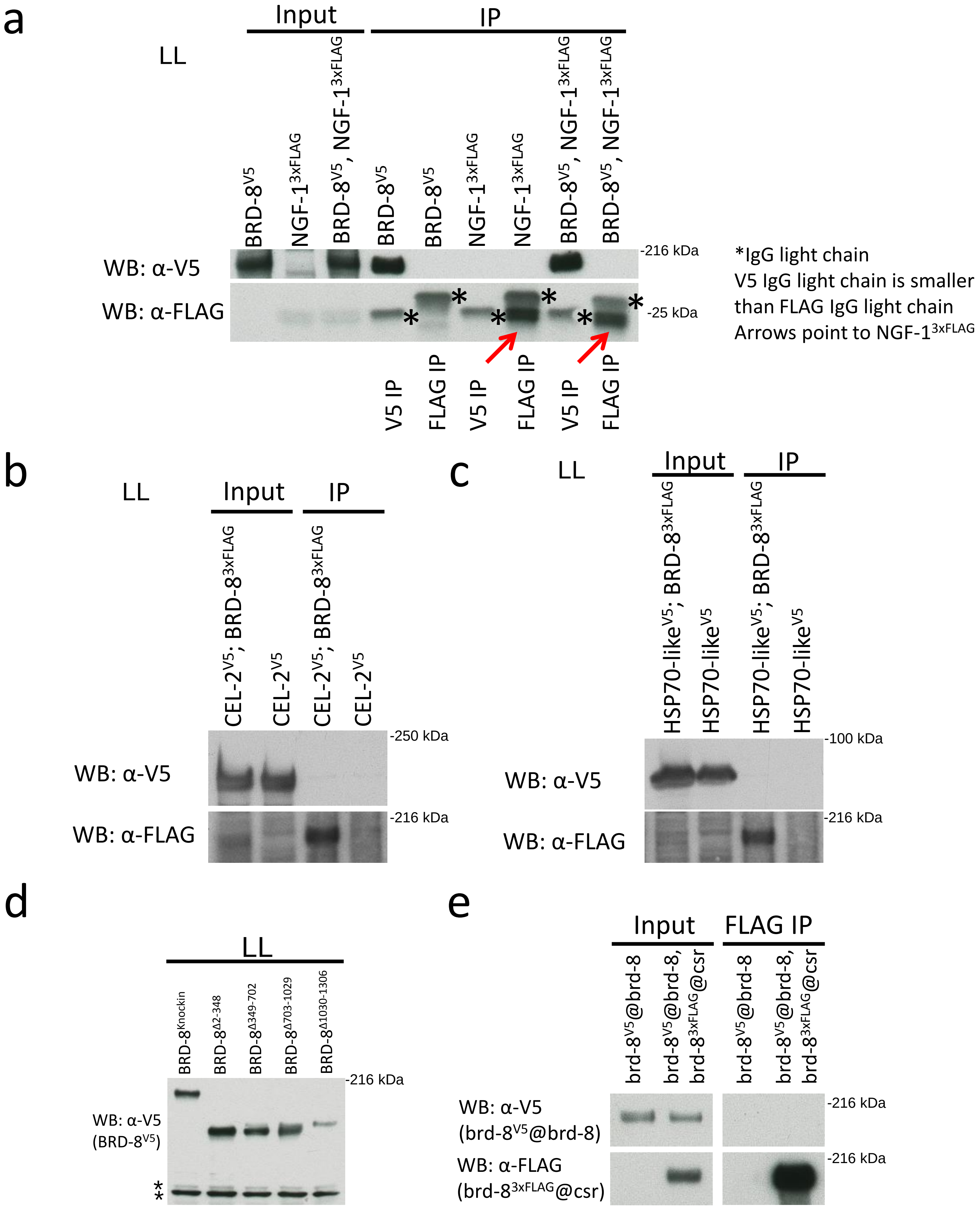

Supplementary Figure 4 continued

Supplementary Figure 4 BRD-8^{V5} does not interact with NGF-1$^{3 \times FLAG}$, CEL-2^{V5}, HSP70-like^{V5}, or itself (**a**) Immunoprecipitation was performed by FLAG or V5 antibody as indicated using BRD-8^{V5}, NGF-1$^{3 \times FLAG}$, and BRD-8^{V5}/ NGF-1$^{3 \times FLAG}$ followed by Western blotting against V5 or FLAG. Asterisks indicate IgG light chains of V5 or FLAG antibody and red arrows point to NGF-1$^{3 \times FLAG}$. (**b**) CEL-2^{V5} (fatty acid synthase beta subunit dehydratase (NCU07307)); BRD-8$^{3 \times FLAG}$ was immunoprecipitated with V5 and Western blotted by V5 and FLAG antibodies; CEL-2^{V5} serves as the negative control for the IP. (**c**) Heat shock protein 70-5 (HSP70-like, NCU08693) in BRD-8$^{3 \times FLAG}$ was tagged with V5, immunoprecipitated with V5 antibody, and Western blotted with V5 and FLAG antibodies. HSP70-like^{V5} is the negative control for the IP. (**d**) Western blotting of *brd-8* deletion strains grown in the light at 25 °C verifying expression levels. (**e**) BRD-8 does not interact with itself. The native BRD-8 was tagged with V5 and a second copy of 3 x FLAG-tagged BRD-8 driven by its native promoter was knocked in the *csr* locus. BRD-8$^{3 \times FLAG}$ was immunoprecipitated by FLAG antibody-conjugated resin and followed by Western blotting with V5 and FLAG antibodies respectively. Experiments in Supplementary Figures 4a-e were repeated twice (n = 3 in total), and similar results as the ones shown here were obtained. Source data except for the blots for Supplementary Figure 4a that were damaged in a lab fire were saved in the Source Data file. The marker position in Supplementary Figure 4a was estimated based on the molecular weight of the mouse immunoglobulin light chain (~25 kDa), which was cross-reacted with the goat anti-mouse secondary antibody (IgG [Immunoglobulin G], HRP (horseradish peroxidase)-conjugated) in Western blotting.

Supplementary Figure 5

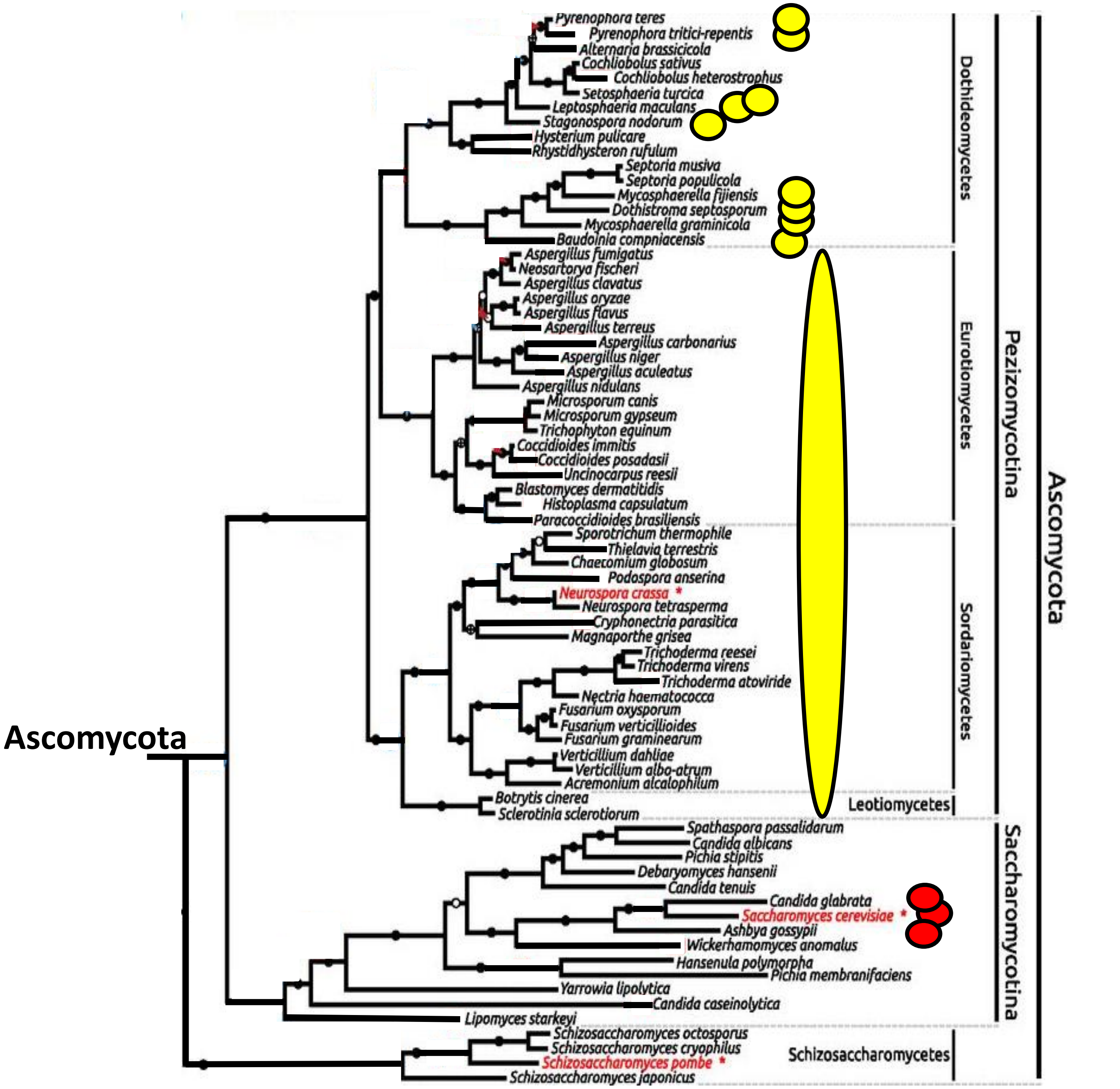

Supplementary Figure 5. Phylogenetic conservation of *Neurospora* BRD-8 and Saccharomyces EAF5. Using a phylogenetic tree adapted from[96], species having orthologs of BRD-8 are marked with yellow circles or ovals, and species with orthologs of EAF5 are marked with red circles. BRD-8 orthologs are universal within sequenced Leutiomycetes, Sordariomycetes, and Eurtiomycetes and common within Dothideomycetes, not all of which have sequenced proteomes available; EAF5 orthologs appear restricted to Saccharomyces and close allies. Orthology was determined by NCBI BLASTP searches as implemented on the SGD website [www.yeastgenome.org] using amino acid sequences for the two proteins.

Supplementary Figure 6

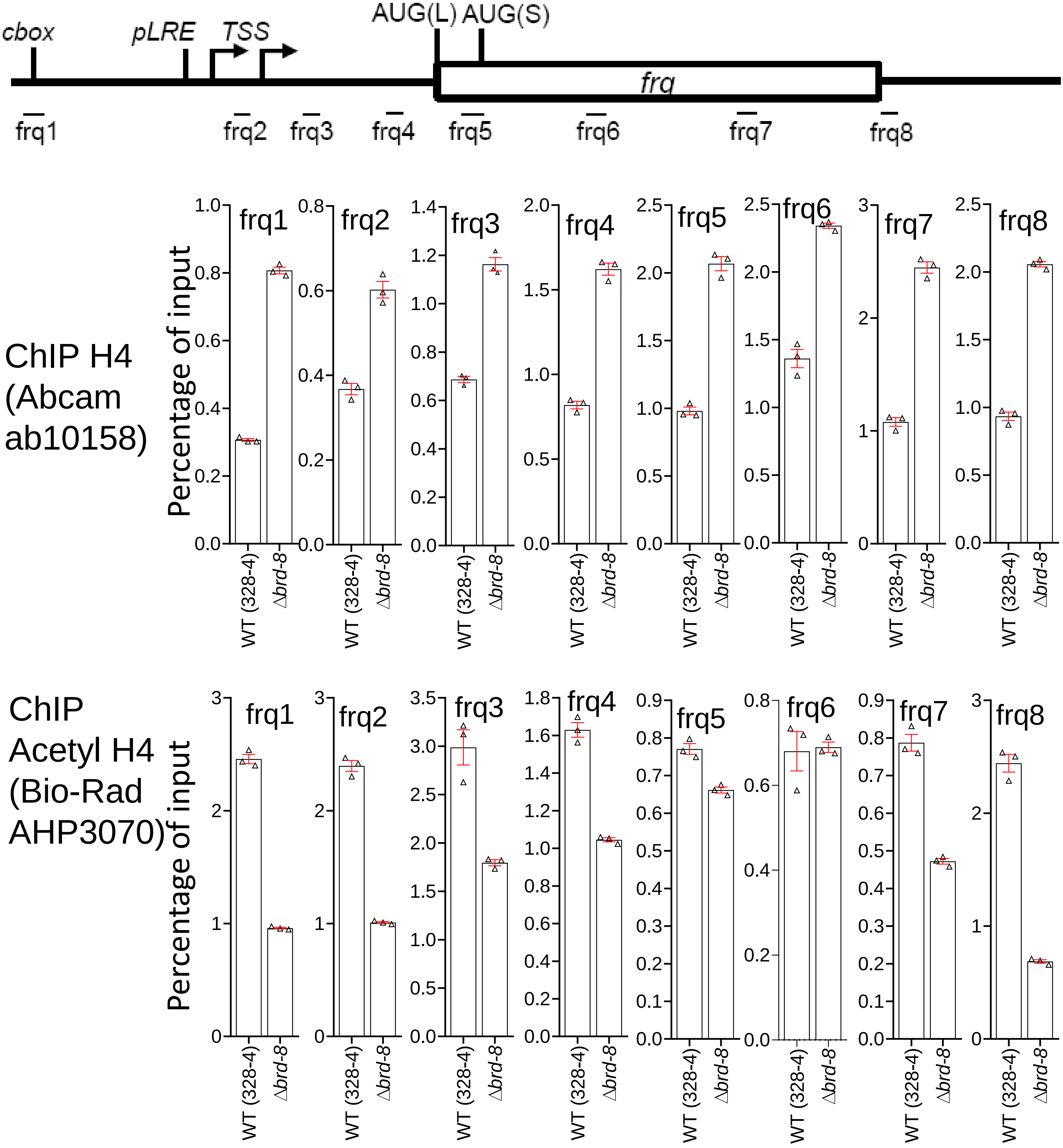

WT (328-4) at DD20 and Δ*brd-8* at DD26 to compensate phase and period differences

Supplementary Figure 6 continued

Supplementary Figure 6. Validation of the ChIP-sequencing data of *frq* in Figure 4a by ChIP quantitative PCR assays. WT and Δ*brd-8* were harvested at DD20 and DD26 respectively after being crosslinked with formaldehyde for 15 min. ChIP experiments were done with antibodies against histone H4 or acetyl histone H4 (at K5, K8, K12, and K16) as indicated. The depiction of primer pairs used in the quantitative PCR reactions here is a copy of the one at the bottom of Figure 4a. See the Source Data file for source data.

Supplementary Figure 7

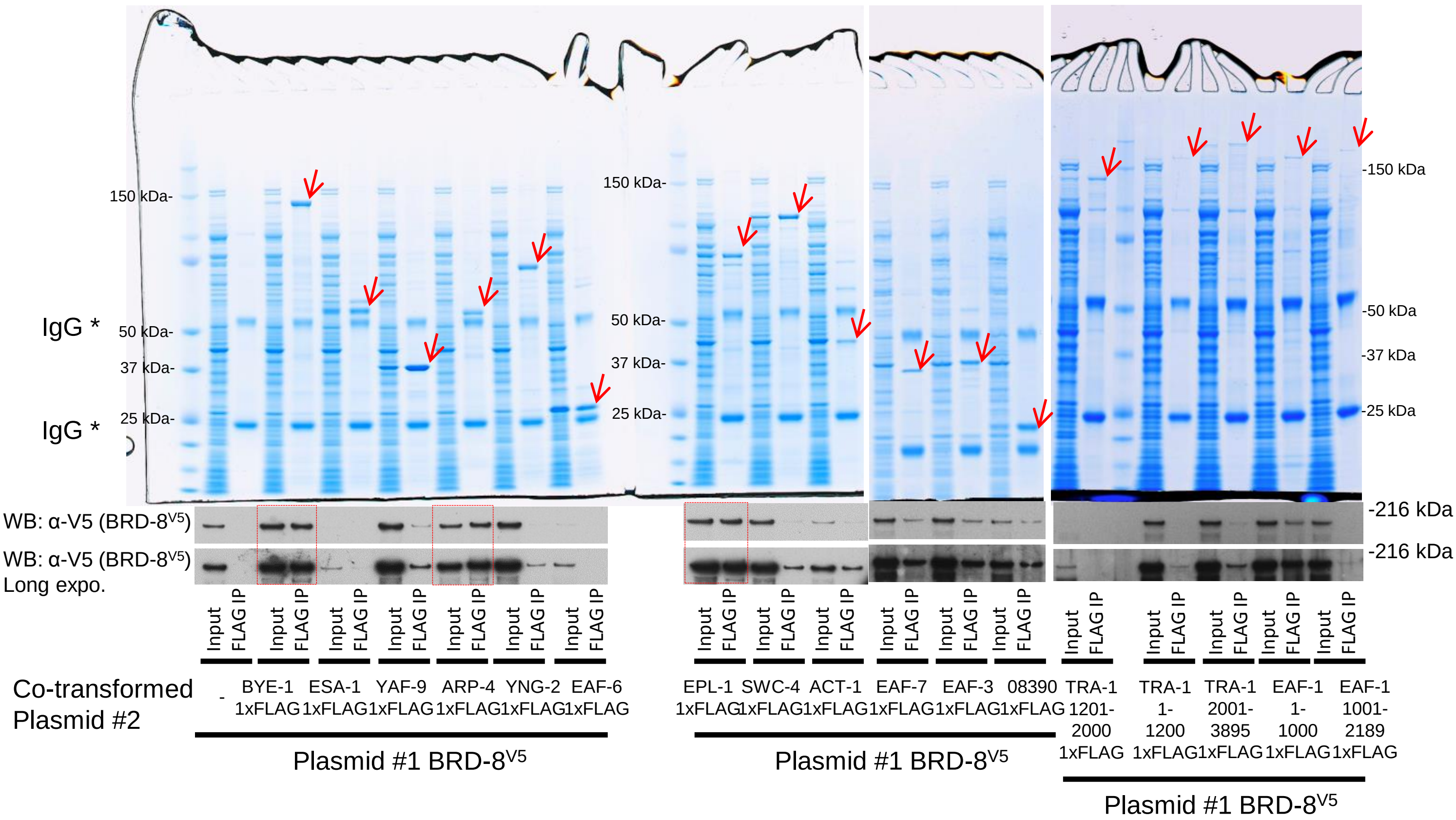

Supplementary Figure 7. Interaction of BRD-8 and NuA4 subunits in bacteria. Plasmids expressing BRD-8^{V5} and BYE-1$^{1 \times FLAG}$ or individual NuA4 subunits as indicated (tagged with 1 x FLAG respectively at their C-termini) were co-transformed to *Escherichia coli* (BL21 DE3), and their interactions were tested by IP with FLAG antibody. Gels were stained with Coomassie blue to show expression of BYE-1$^{1 \times FLAG}$ and individual NuA4 subunits, and Western blotting was performed with V5 to display expression and interaction levels of BRD-8^{V5} with these proteins individually. Red arrows point to protein bands obtained from IPs with FLAG resin with the expected sizes; two exposures of V5 blots were shown to better visualize and compare BRD-8^{V5} levels in inputs and FLAG IPs. The assay in Supplementary Figure 7 was repeated three times independently with similar observations. Source data were included in the Source Data file.

Supplementary Figure 8

a

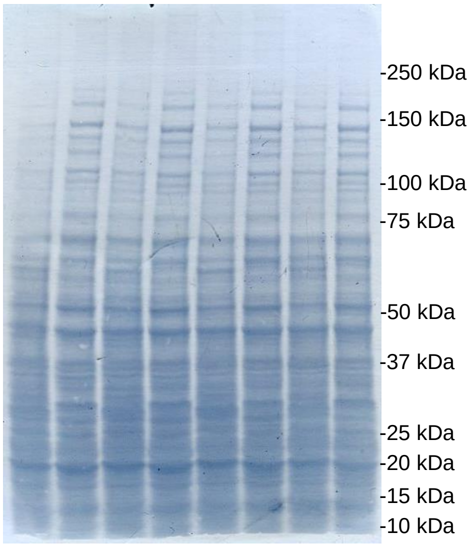

b

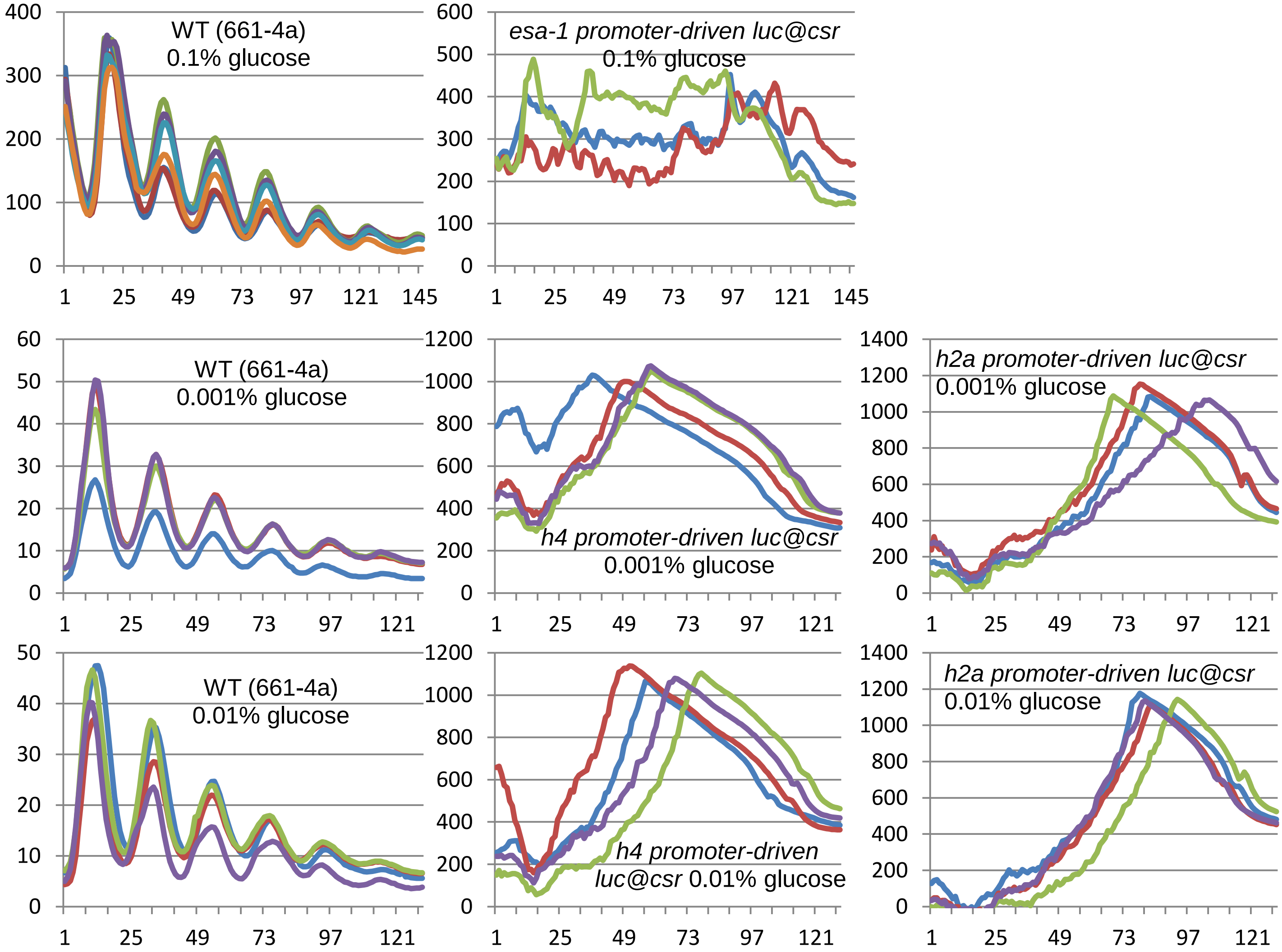

Supplementary Figure 8 continued

Supplementary Figure 8 Promoter activity of *esa-1*, *histone h4*, or *histone h2a* in the dark. Blot staining for figure 6d (**a**) and luciferase analyses of the promoter of *esa-1*, *histone h4*, or *histone h2a* driving the *luciferase* gene at the *csr* locus (**b**). The assay was performed at 0.1, 0.01, or 0.001% of glucose in the culturing medium as indicated. The experiment in Supplementary Figure 8a was performed three times with similar results. Source data can be retrieved from the Source Data file.
