# Supplementary material for: A crucial role for dynamic expression of components encoding the negative arm of the circadian clock
Source: Nat Commun. 2023 Jun 8;14:3371. doi: 10.1038/s41467-023-38817-7 (PMC10250352; doi:10.1038/s41467-023-38817-7)
Supplement: Supplementary file 3 — Description of Additional Supplementary Files [file 41467_2023_38817_MOESM3_ESM.pdf]

Description of Additional Supplementary Files

File Name: Supplementary Data 1
Description: BRD-8^{V5} interactome identified by mass spectrometry. Cleared lysate from WT or BRD-8^{V5} was immunoprecipitated with V5 antibody-conjugated Dynabeads and the immunoprecipitated analyzed by mass spectrometry (See Methods for details).

File Name: Supplementary Data 2
Description: Densities of pan-histone H4 and acetyl histone H4 in WT at D20 hrs and Δ*brd-8* at D26 hrs. The six-hr lag between WT and Δ*brd-8* accounts for their period and phase differences between the two strains. The peak calling/comparison software SICER v1.1[97] was used to assess differences in comparable peaks between WT and mutant samples. SICER assesses departure from a Poisson distribution to determine up- and down-regulated differences in peak size using separate one-sided tests; then an FDR correction is applied. Descriptions in details for the experiment can be found in Methods
