# Supplementary material for: A crucial role for dynamic expression of components encoding the negative arm of the circadian clock
Source: Nat Commun. 2023 Jun 8;14:3371. doi: 10.1038/s41467-023-38817-7 (PMC10250352; doi:10.1038/s41467-023-38817-7)
Supplement: Supplementary file 6 — Reporting Summary [file 41467_2023_38817_MOESM6_ESM.pdf]

nature portfolio

Corresponding author(s): Jay Dunlap

Last updated by author(s): May 17, 2023

Reporting Summary

Nature Portfolio wishes to improve the reproducibility of the work that we publish. This form provides structure for consistency and transparency in reporting. For further information on Nature Portfolio policies, see our Editorial Policies and the Editorial Policy Checklist.

Statistics

For all statistical analyses, confirm that the following items are present in the figure legend, table legend, main text, or Methods section.

n/a	Confirmed	
☐	☒	The exact sample size (*n*) for each experimental group/condition, given as a discrete number and unit of measurement
☐	☒	A statement on whether measurements were taken from distinct samples or whether the same sample was measured repeatedly
☐	☒	The statistical test(s) used AND whether they are one- or two-sided<br>*Only common tests should be described solely by name; describe more complex techniques in the Methods section.*
☒	☐	A description of all covariates tested
☒	☐	A description of any assumptions or corrections, such as tests of normality and adjustment for multiple comparisons
☐	☒	A full description of the statistical parameters including central tendency (e.g. means) or other basic estimates (e.g. regression coefficient) AND variation (e.g. standard deviation) or associated estimates of uncertainty (e.g. confidence intervals)
☐	☒	For null hypothesis testing, the test statistic (e.g. *F*, *t*, *r*) with confidence intervals, effect sizes, degrees of freedom and *P* value noted<br>*Give P values as exact values whenever suitable.*
☒	☐	For Bayesian analysis, information on the choice of priors and Markov chain Monte Carlo settings
☒	☐	For hierarchical and complex designs, identification of the appropriate level for tests and full reporting of outcomes
☒	☐	Estimates of effect sizes (e.g. Cohen's *d*, Pearson's *r*), indicating how they were calculated

Our web collection on statistics for biologists contains articles on many of the points above.

Software and code

Policy information about availability of computer code

Data collection	Torrent Suite TM Software (Ver. 4.0.2); TMAP (Ion Software, https://github.com/iontorrent/TMAP); htseq-qa (http://htseq.readthedocs.io/en/release_0.9.1/); MACS2 (https://github.com/taoliu/MACS); ChIPseeker (Bioconductor, https://bioconductor.org/packages/release/bioc/html/ChIPseeker.html)
Data analysis	SICER software (https://home.gwu.edu/~wpeng/Software.htm)

For manuscripts utilizing custom algorithms or software that are central to the research but not yet described in published literature, software must be made available to editors and reviewers. We strongly encourage code deposition in a community repository (e.g. GitHub). See the Nature Portfolio guidelines for submitting code & software for further information.

Data

Policy information about availability of data

All manuscripts must include a data availability statement. This statement should provide the following information, where applicable:

- Accession codes, unique identifiers, or web links for publicly available datasets
- A description of any restrictions on data availability
- For clinical datasets or third party data, please ensure that the statement adheres to our policy

The mass spectrometry and ChIP-seq data generated in this study have been deposited in the MassIVE database under accession: MSV000089415 (link: https://

massive.ucsd.edu/ProteoSAFe/dataset.jsp?task=74b7985c9ee9409485fb212a918c6b4b, PDX#: PXD033576) and NCBI SRA under accession: PRJNA834768 (https://www.ncbi.nlm.nih.gov/sra/PRJNA834768), respectively.

Human research participants

Policy information about studies involving human research participants and Sex and Gender in Research.

Reporting on sex and gender	This research involved no human subjects.
Population characteristics	This research involved no human subjects.
Recruitment	This research involved no human subjects.
Ethics oversight	This research involved no human subjects.

Note that full information on the approval of the study protocol must also be provided in the manuscript.

Field-specific reporting

Please select the one below that is the best fit for your research. If you are not sure, read the appropriate sections before making your selection.

☒ Life sciences ☐ Behavioural & social sciences ☐ Ecological, evolutionary & environmental sciences

For a reference copy of the document with all sections, see nature.com/documents/nr-reporting-summary-flat.pdf

Life sciences study design

All studies must disclose on these points even when the disclosure is negative.

Sample size	Samples for WB were collected every four hours in the dark or as indicated, which follows conventions in Neurospora circadian research. ChIP-seq or ChIP-PCRs were performed with WT collected at D20 while brd-8 KO at D26. The detailed information can be found in figure legends.
Data exclusions	No data was excluded in this research.
Replication	Luciferase assays were performed with three replicates, RT-qPCR had three or more biological replicates as noted, and race tubes three or more technical replicates as noted. All attempts at replication were successful. Other than replications mentioned here, no other replications were done.
Randomization	Randomization is not a component in our experiment design, because we focused on the function of one gene, examining biochemical, genomic, and gene-expression effects when it is absent.
Blinding	Blinding is not required for this research, which focuses on functions of a single gene by means of biochemical and molecular-biology analyses. Results are not drawn on random sampling and relevant statistics.

Reporting for specific materials, systems and methods

We require information from authors about some types of materials, experimental systems and methods used in many studies. Here, indicate whether each material, system or method listed is relevant to your study. If you are not sure if a list item applies to your research, read the appropriate section before selecting a response.

Materials & experimental systems

n/a	Involved in the study
☐	☒ Antibodies
☒	☐ Eukaryotic cell lines
☒	☐ Palaeontology and archaeology
☒	☐ Animals and other organisms
☒	☐ Clinical data
☒	☐ Dual use research of concern

Methods

n/a	Involved in the study
☐	☒ ChIP-seq
☒	☐ Flow cytometry
☒	☐ MRI-based neuroimaging

Antibodies

Antibodies used	Antibodies against WC-1 (1:250, Lee et al., 2000 Science), WC-2 (1:5,000, Denault et al., 2001 EMBO J), FRQ (1:250, Garceau et al., Cell), and FRH (1:5,000, Shi et al. 2010 Genetics) have been described previously. Other antibodies and antibody-related products used in this study include V5 antibody (1:5,000, Thermo Pierce, Catalog # 46-0705), FLAG antibody (1:5,000, Sigma-Aldrich, Catalog # F1804-1MG), HA (1:5000, Abcam, Catalog # ab9110), V5 agarose (25 uL:2 mg lysate, Sigma-Aldrich, Catalog # 7345), FLAG M2 resin (25 uL: 2 mg lysate, Sigma-Aldrich, Catalog # A2220), V5 antibody (1:10,000, Abcam, Catalog # ab9116), rabbit anti-acH4 (1:10,000, UBI, Catalog # 06–866), and anti-acH2A K9 (1:1,000, Millipore Sigma, Catalog # 07-289).
Validation	WC-1 (produced in rabbit for WB [Lee et al., 2000 Science]), WC-2 (produced in rabbit for WB [Denault et al., 2001 EMBO J]), FRQ (produced in rabbit for WB [Garceau et al., 1997 cell]), and FRH (produced in rabbit for WB [Shi et al. 2010 Genetics])antibodies have been validated in previous publications, and commercial antibodies have been validated by sellers and product reviews including publications. V5 (Thermo): https://www.thermofisher.com/antibody/product/V5-Tag-Antibody-clone-SV5-Pk1-Monoclonal/R960-25; FLAG: https://www.sigmaaldrich.com/US/en/product/sigma/f1804; HA: https://www.abcam.com/products/primary-antibodies/ha-tag-antibody-chip-grade-ab9110.html; V5 agarose: https://www.sigmaaldrich.com/US/en/product/sigma/a7345; FLAG M2 resin: https://www.sigmaaldrich.com/US/en/product/sigma/a2220; V5 (Abcam): https://www.abcam.com/products/primary-antibodies/v5-tag-antibody-ab9116.html; AcH4: https://www.sigmaaldrich.com/US/en/product/mm/06866; AcH2A: https://www.sigmaaldrich.com/US/en/product/mm/07289

ChIP-seq

Data deposition

☐ Confirm that both raw and final processed data have been deposited in a public database such as GEO.

☐ Confirm that you have deposited or provided access to graph files (e.g. BED files) for the called peaks.

Data access links<br>*May remain private before publication.*	https://www.ncbi.nlm.nih.gov/sra/PRJNA834768<br>https://www.ncbi.nlm.nih.gov/geo/query/acc.cgi?acc=GSE216517
Files in database submission	Six files: ChIPAcH4, ChIPH4, Input, ChIPAcH4, ChIPH4, Input
Genome browser session<br>(e.g. UCSC)	The Integrative Genomics Viewer (IGV)

Methodology

Replicates	The ChIP-seq result provided qualitative information and then validated by ChIP-qPCR
Sequencing depth	328_4D20H4.fastq 14.48x<br>328_4D20H4AC.fastq 20.13x<br>328_4D20_Input.fastq 19.26x<br>Delta82D26H4.fastq 20.52x<br>Delta82S26H4AC.fastq 15.39x<br>Delta82D26_Input.fastq 12.17x
Antibodies	Anti-Histone H4 antibody - ChIP Grade (Abcam, Catalog # ab7311) and anti-acH4 antibody(UBI, Catalog # 06–866)
Peak calling parameters	Peaks were called using MACS2(https://github.com/taoliu/MACS) with the--broad and --nomodel settings used.
Data quality	Mapping efficiency and read quality were visualized using htseq-qa (http://htseq.readthedocs.io/en/release_0.9.1/).
Software	TMAP (Ion Software, https://github.com/iontorrent/TMAP); MACS2 (https://github.com/taoliu/MACS).
